# Phylogeography of *Dictyota fasciola* and *Dictyota mediterranea* (Dictyotales, Phaeophyceae): unexpected patterns on the Atlantic-Mediterranean marine transition and taxonomic implications

**DOI:** 10.7717/peerj.6916

**Published:** 2019-05-16

**Authors:** Daniel Vitales, Joana Aragay, Teresa Garnatje, Amelia Gómez Garreta, Jordi Rull Lluch

**Affiliations:** 1 Institut Botànic de Barcelona (IBB, CSIC-ICUB), Barcelona, Catalonia, Spain; 2 Laboratori de Botànica, Facultat de Farmàcia i Ciències de l’Alimentació & Institut de Recerca de la Biodiversitat (IRBio), Universitat de Barcelona, Barcelona, Catalonia, Spain

**Keywords:** Algae, Biogeography, *Cox1*, Genetic diversity, Haplotype, Messinian salinity crisis, Pleistocene glaciations, *rbc*L-*rbc*S intergenic spacer, Refugia, Seaweeds

## Abstract

The Atlantic-Mediterranean marine transition is a fascinating biogeographic region, but still very poorly studied from the point of view of seaweed phylogeography. *Dictyota fasciola* and *D. mediterranea* (Dictyotales, Phaeophyceae) are two currently recognized sister species that share a large part of their distribution along the Mediterranean Sea and the Atlantic Ocean, representing a unique study model to understand the diversification processes experienced by macroalgae during and after Messinian at this marine region. In this study, we sampled 102 individuals of *D. fasciola* and *D. mediterranea* from 32 localities along their distribution range and sequenced the mitochondrial *cox*1 and the chloroplast *rbc*L*-rbc*S DNA regions for all the samples. Our data do not support the occurrence of two sister species but a morphologically variable and highly genetic diverse species or a complex of species. Most of the observed genetic diversity corresponds to the Mediterranean populations, whereas the Atlantic ones are much more homogeneous. The early-diverged lineages inferred from both mtDNA and cpDNA phylogenetic reconstructions were constituted by samples from the Mediterranean Sea. Together, these results suggest that the Mediterranean Sea acted as a refugium for the *D. fasciola*–*D. mediterranea* lineage during the geologic and climatic changes occurred on the region since the Miocene, subsequently dispersing to the Atlantic Ocean.

## Introduction

In the last decades, the increase of DNA sequencing data has been a key step to achieve a better understanding of biodiversity, constituting the basis of modern fields like integrative taxonomy and molecular systematics (Dayrat, 2005; Will, Mishler & Wheeler, 2005; Hajibabaei et al., 2007; Maddison, Schulz & Maddison, 2007; Schlick-Steiner et al., 2010). This source of information is particularly important for improving our knowledge of organisms such as macroalgae, frequently showing poor diagnostic phenotypical characters (Verbruggen, 2014). In this way, many studies based on DNA have contributed to recognizing phenotypically cryptic seaweed species (Leliaert et al., 2014 and references therein) or to redefining classifications of some lineages, establishing evolutionarily natural groups (Brodie & Lewis, 2007 and references therein). The advances in these fields also served as a basis to phylogeography, a discipline where seaweeds have experienced increasing relevance during recent years (Hu, Duan & Lopez-Bautista, 2016).

Comparative phylogeography on diverse marine organisms has demonstrated to be a useful tool to unravel evolutionary and ecological patterns across marine provinces and biodiversity hotspots (Bowen et al., 2016). However, the relevance of seaweed studies on some geographical regions such as the Atlantic-Mediterranean transition is still very poor compared to other organisms like animals or land plants (Patarnello, Volckaert & Castilho, 2007; Hu, Duan & Lopez-Bautista, 2016). For instance, most data concerning the diversification processes on this region during the key Miocene-Pliocene boundary come from marine animals (e.g., crustacean Rastorgueff et al., 2014; echinoderms, Taboada & Pérez-Portela, 2016; or vertebrates, (Valsecchi et al., 2005. According to the most accepted hypothesis, no true marine organisms could have survived in the brackish-water or hypersaline lakes that remained in the Mediterranean Basin during the Messinian Salinity Crisis (MSC; 7.25–5.33 Ma) (Taviani, 2002). Consequently, the Mediterranean Sea would have been recolonized by species occurring in the Atlantic Ocean following the flooding after the MSC (Hsü et al., 1977). In contrast, other studies suggest some true marine enclaves persisted in the deeper areas of the Mediterranean and served as refugia for some “Messinian” species (e.g., Boudouresque, 2004; Sotelo, Morán & Posada, 2009; Reuschel, Cuesta & Schubart, 2010).

Climatic changes during Plio-Pleistocene also had a great impact on the Atlantic-Mediterranean marine transition and the organisms inhabiting this region (Patarnello, Volckaert & Castilho, 2007). Several investigations have reported that latitudinal and sea-level shifts associated with Pleistocene glacial-interglacial cycles fuelled important range changes and vicariance events on Atlantic-Mediterranean marine protists (e.g., Lowe et al., 2012), animals (e.g., Xavier et al., 2011) and seagrasses (e.g.,  Arnaud-Haond et al., 2007; Alberto et al., 2008). To our knowledge, the only phylogeographic study involving a native seaweed from the Mediterranean Sea focuses on the red coralline algae *Lithophyllum byssoides* (Lamarck) Foslie (Pezzolesi et al., 2017). Based on the genetic differences found among Atlantic and Mediterranean specimens, the authors suggested that MSC and Plio-Pleistocene climatic changes shaped genetic structure of this species. However, the sampling of the study—restricted to the central Mediterranean populations (Ligurian, Tyrrhenian and Adriatic Seas) plus two Atlantic specimens from the Iberian Peninsula—limited the inference of further phylogeographic patterns.

*Dictyota fasciola* (Roth) J.V.Lamouroux is a relatively common species of eulittoral pools and the shallow subtidal zones in the NE Atlantic and the Mediterranean Sea. *Dictyota mediterranea* (Schiffner) G.Furnari is a rarer species, endemic to the coasts of the Mediterranean Sea where it occupies a similar habitat to that of the preceding species. As occurs in the majority of *Dictyota* species, these two taxa are notoriously difficult to identify based on morphological, anatomical, or reproductive characters. In this way, *D. mediterranea* was formerly reduced to a synonym of *D. fasciola* by Feldmann (1937) on the basis of similarities in colour, width of the axes, and shape of the apices. However, subsequent authors recognise *D. mediterranea* as a different species (Coppejans, 1983; Ribera et al., 1992; Pena Martín, Gómez Garreta & Crespo, 2004; Cormaci et al., 2012; Guiry & Guiry, 2019). Indeed, *D. mediterranea* shows a terete thallus at the base and the apex—but complanate in the middle part—and a multilayered medulla; whereas axes of *D. fasciola* are all complanate, and a multilayered medulla is restricted to the basal parts of the thallus (Pena Martín, Gómez Garreta & Crespo, 2004; Cormaci et al., 2012). Previous molecular phylogenetic studies indicated that these species are closely related (Tronholm et al., 2010), but results of the same study pointed out a noticeable genetic differentiation among them. The divergence between *D. fasciola* and *D. mediterranea* was estimated to occur c. 6.5 Ma (10 Ma–4 Ma; 95% highest density probability) according to a time calibrated multigene phylogeny of the genus *Dictyota* (Tronholm et al., 2012), partially overlapping with the start of the MSC (Krijgsman et al., 1999). Based on these former data, Tronholm et al. (2010) speculated that *D. fasciola*–*D. mediterranea* lineage would have an Atlantic origin, subsequently colonizing the Mediterranean basin—either before or after the divergence of both species—after the MSC. As the only example of two sister *Dictyota* species occurring along the Mediterranean Sea and the Atlantic Ocean, these two taxa represent a unique study model to understand the phylogeographic processes experienced by macroalgae during and after Messinian at this marine region.

In this study, we use a broad sampling along the distribution range of these *Dictyota* species to investigate their diversification process. Based on the sequences obtained from two variable mitochondrial (*cox*1) and chloroplast (*rbc*L-*rbc*S) DNA regions, we address three main goals. First, we aim to validate the taxonomic differentiation among *D. fasciola* and *D. mediterranea* observed in previous phylogenetic studies of the genus. Second, we will test whether our phylogeographic data fit well to the former “Atlantic to Mediterranean” colonization hypothesis proposed to explain the evolutionary history of this lineage. Finally, we discuss the contribution of our results to the knowledge about the Atlantic-Mediterranean transition during the Messinian and the Plio-Pleistocene periods.

**Table 1:** Summary of sampling locations, geographic circumscription, number of individuals (N) and haplotype information of *D. fasciola* and *D. mediterranea* specimens used in this study.

Taxonomic assignation	Geographic region	Sampling site	Code	N	mtDNA haplotype	cpDNA haplotype
*Dictyota fasciola*	WM	Spain: Alicante, Cabo de Huertas	F-Alac	2	M1	C1/C5
		Spain: Almería, La Isleta	F-Isle	4	M6(1) ,M18(3)	C12(3)/C13(1)
		Spain: Catalonia, Llançà	F-Llan	5	M1(2), M4(1), M5(1), M7(2)	C1(1), C3(1), C4(2), C8(1)
		Spain: Castellò, Serra d’Irta	F-Cast	6	M1(3),M7(3)	C1(3)/C5(3)
		France: Côte Vermeille, Cerbère	F-Cerb	5	M12	C1
		France: Côte Vermeille, Banyuls-sur-mer	F-Bany	5	M1(2),M12(1),M15(2)	C1
		France: Nice	F-Nice	5	M1(1),M12(4)	C1
		Italy: Sardegna, Isola Rosa	F-SaIR	2	M14	C1(1)/C11(1)
		Italy: Sardegna, Porto Ferro	F-SaPF	4	M12(1),M17(2),M20(1)	C1(1)/C11(3)
	EM	Greece: Central Macedonia	F-CeMa	2	M1(1)M16(1)	C1(1)/C15(1)
		Greece: Karpathos, Agios Nikolaos	F-Karp	2	M1(1),M19(1)	C7(1)/C10(1)
		Greece: Rhodes, Ladiko Bay	F-RhoL	2	M19	C5(1)/C10(1)
		Greece: Rhodes, Fourni	F-RhoF	1	M9	C7
		Italy: Sicily, Aci Castello	F-Sici	1	M11	C1
	ATL	Portugal: Porto Covo	F-Port	3	M1	C1
		Portugal: Madeira, Ponta do Sao Lourenço	F-MaPo	1	M2	C1
		Portugal: Madeira, Reis Magos	F-MaRe	1	M1	C1
		Spain: Cádiz, Tarifa	F-Tari	5	M1	C1
		Spain: Canary Is., Lanzarote, Famara	F-LaFa	1	M1	C1
		Spain: Canary Is., Lanzarote, Puerto del Carmen	F-LaPC	1	M1	C1
		Spain: Canary Is., La Graciosa	F-Grac	1	M1	C1
		Spain: Canary Is., Gran Canaria, Medio Almud	F-GCMA	1	M1	C1
		Spain, Canary Is., Gran Canaria, Maspalomas	F-GCPM	1	M1	C1
		Spain: Canary Is., Tenerife, Punta Hidalgo	F-TePH	3	M1	C1(2)/C2(1)
		Spain: Canary Is., Tenerife, Buenavista	F-TeBu	1	M1	C1
		Spain: Canary Is., El Hierro	F-ElHi	2	M1(1),M3(1)	C1
*Dictyota mediterranea*	WM	Spain: Alacant, Cabo de Huertas	M-Alac	4	M7(2),M10(2)	C5
		Spain: Mallorca, Alcúdia	M-Mall	1	M7	C5
		Spain: Almería, La Isleta	M-Isle	2	M10	C5
		Spain: Catalonia, Llançà	M-Llan	9	M16	C14(3),C15(6)
		France: Côte Vermeille, Banyuls-sur-mer	M-Bany	8	M21(6),M22(1),M12(1)	C1(1),C16(6),C17(1)
		Italy: Sicily, Capo di Milazzo	M-SiCM	1	M7	C5
	EM	Italy: Sicily, Giardini Naxos	M-SiGN	1	M13	C18
		Italy: Sicily, Aci Castello	M-SiCi	2	M7	C5
		Greece: Rhodes, Ladiko Bay	M-RhoL	3	M1(1), M7(2)	C5
		Greece: Rhodes, Agios Thomas	M-RhoA	2	M9	C5(1),C6(1)
		Greece: Karpathos, Kastellia Bay	M-KarK	1	M8	C9
		Greece: Karpathos, Christou Pigadi	M-KarC	1	M8	C5

## Materials & Methods

### Sampling and sequencing

**Figure 1:**
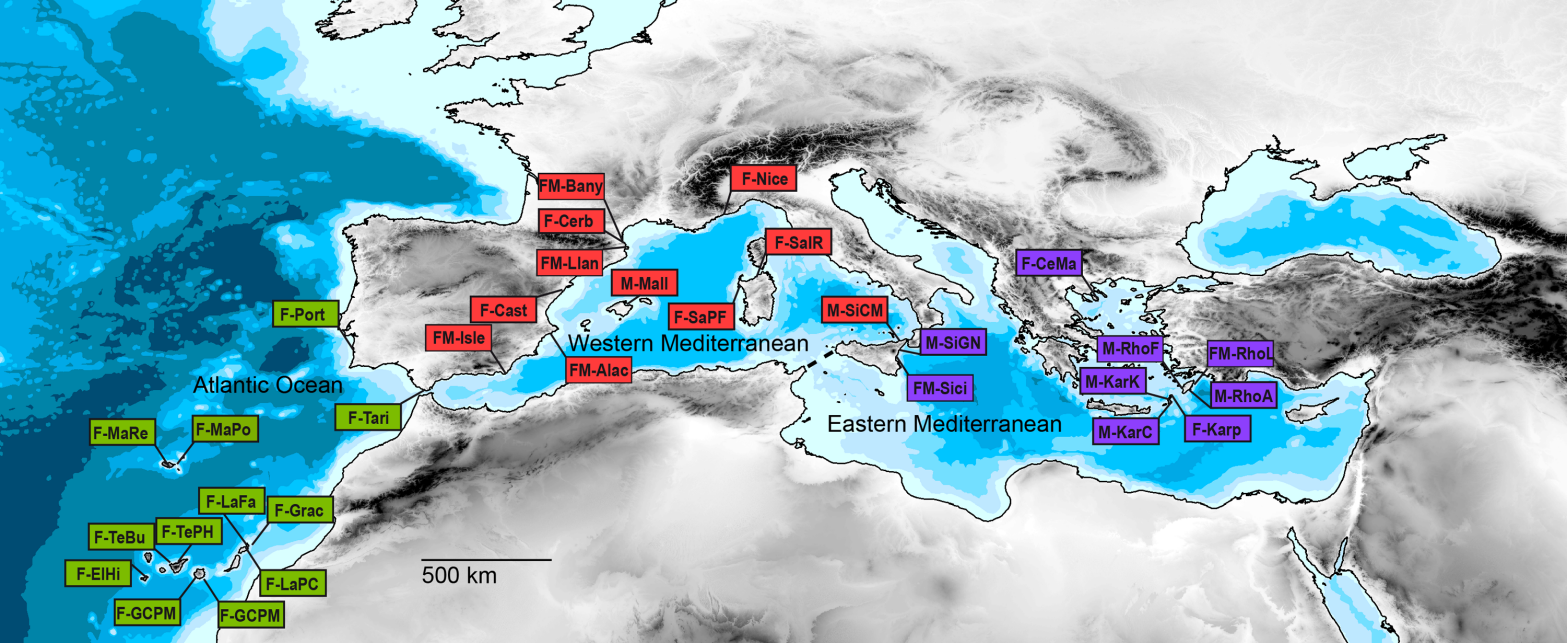
Geographic distribution of the samples analyzed in this study (sample code according to Table 1). The color of the square indicates the geographic circumscription to three main biogeographic marine regions (i.e., Atlantic Ocean, in green; Western Mediterranean Sea, in red; and Eastern Mediterranean Sea, in violet).

We sampled 102 individuals of *D. fasciola* (67 specimens) and *D. mediterranea* (35 specimens) from 32 sampling sites along their main distribution range (see Tronholm et al., 2010) in the Mediterranean Sea and the Atlantic Ocean (Table 1; Fig. 1). Specimens were identified first in the field and later in the laboratory. Representative samples from all localities were preserved on herbarium sheets and deposited in the BCN-Phyc (Centre de Documentació de Biodiversitat Vegetal, Universitat de Barcelona, Spain) and GENT (Ghent University, Belgium) herbaria. Geographic coordinates for each sampling site are shown in Table S1. The CTAB method (Doyle & Doyle, 1987) with modifications (Soltis et al., 1991; Cullings, 1992) was used to extract total genomic DNA from silica-dried material derived from fresh tissue. The mitochondrial *cox*1 and the chloroplast *rbc*L*-rbc*S regions were amplified and sequenced for all the samples. Amplification procedure was performed as described in Aragay et al. (2017). Direct sequencing of the amplified DNA segments was performed with Big Dye Terminator Cycle Sequencing v 3.1 (PE Biosystems, Foster City, California, USA) at the Unitat de Genòmica, Centres Científics i Tecnològics, Universitat de Barcelona (CCiTUB) on an ABI PRISM 3700 DNA analyser (PE Biosystems). The sequencing primers used were the same as the amplification ones. Sequences were edited and assembled using Chromas Lite v 2.01 (Technelysium PTy, Tewantin, Queensland, Australia) and Bioedit v 7.0.9 (Ibis Biosciences, Carlsbad, CA, USA). The alignment was conducted in Clustal W (Thompson, Higgins & Gibson, 1994) and finally adjusted by hand. GenBank accession numbers are provided in Table S1.

### Phylogenetic analyses of *D. fasciola* and *D. mediterranea*

A first molecular phylogenetic reconstruction within the *D. fasciola—D. mediterranea* group was performed by Bayesian inference (BI) with MrBayes v 3.2 (Ronquist et al., 2012), independently for both chloroplast and mitochondrial markers. *Dictyota guineënsis* (Kützing) P.Crouan & H.Crouan was chosen as outgroup according to unpublished phylogenetic analyses at the genus level (Olivier de Clerck, Ghent University, pers. comm.). Despite *Dictyota* presents maternal inheritance for chloroplast and mitochondrial organelles (Motomura, Nagasato & Kimura, 2010), we analysed separately both datasets since their DNA loci may undergo independent evolution, potentially generating incorrect phylogenetic inferences based on concatenated datasets (Degnan & Rosenberg, 2009). Partitioning strategies and models of molecular evolution were selected with Partitionfinder v 2.1.1 (Lanfear et al., 2016). A partitioning scheme with 3 partitions organized by codon position was chosen for the mitochondrial genic region *cox*1 (SYM+G, HKY and HKY+G models for the *cox*1 first, second and third positions, respectively), while one single partition (HKY+G model) was applied for the chloroplast *rbc*L*-rbc*S intergenic spacer. Two independent Markov chain Monte Carlo (MCMC) analyses with four Metropolis-coupled chains each were run for 10 million generations, sampling every 1,000 generations. The first 25% of the trees were discarded as “burn-in”, after confirming that the average standard deviation of the split frequencies was <0.01, and the potential scale reduction factor approached 1.0 for all parameters. The remaining trees were pooled to construct 50% majority-rule consensus trees that approximate the posterior distribution of the phylogenetic reconstructions, and to obtain Bayesian posterior probabilities.

A Maximum Likelihood (ML) approach was also performed using RAxML-HPC v.8 (Stamatakis, 2014), partitioning the datasets as in the Bayesian analysis. Given that RAxML allows for only a single model of rate heterogeneity in partitioned analyses and following the recommendations by Stamatakis (2006), we employed the GTRCAT nucleotide substitution model for all partitions, with the default settings for the optimisation of individual per site substitution rates. The best-scoring ML tree with clade support values was obtained from 10 independent runs, with 1,000 rapid bootstrap replicates each run. Both phylogenetic analyses were performed within the CIPRES Science Gateway (Miller, Pfeiffer & Schwartz, 2010), and the resulting summary trees were visualised in FigTree v.1.4.2 (https://github.com/rambaut/figtree).

### Genetic variability of *D. fasciola* and *D. mediterranea*

For analyses taking into account phylogeographic structuring of populations, the samples were assigned to three main biogeographic marine regions (i.e., Atlantic Ocean, West Mediterranean and East Mediterranean; (Coll et al., 2010). Haplotype minimum-spanning networks (Bandelt, Forster & Röhl, 1999) were reconstructed using PopArt (Leigh & Bryant, 2015), independently for each marker under study, using default settings (i.e., parameter ε = 0) to consider multifurcations and/or reticulations in a phylogenetic network approach.

Haplotype (Hp) and nucleotide (p) diversities were calculated separately for each marker using DnaSP v 5.0 (Rozas & Rozas, 1995). Haplotype richness (R(n)) was computed with RAREFAC (Petit, El Mousadik & Pons, 1998) a software that uses a rarefaction approach to standardize the haplotype richness to a fixed sample size to facilitate comparisons across groups of samples. In this case, the rarefaction value (*n* = 18) was set according to the sample size of the smallest group of populations (i.e., East Mediterraean group).

## Results

### Phylogenetic analyses of *D. fasciola* and *D. mediterranea*

**Figure 2:**
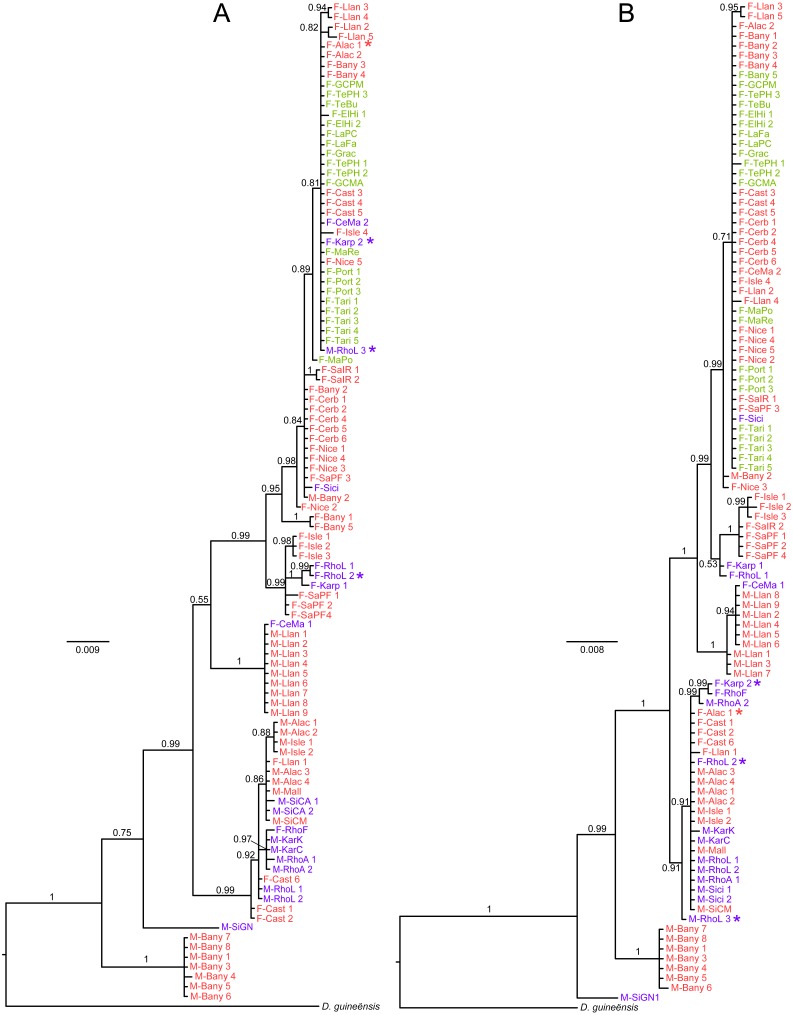
Consensus tree based on Bayesian inference of (A) the mitochondrial *cox*1 region and (B) the chloroplast *rbc*L-*rbc*S intergenic spacer. The color of the labels indicates their geographic origin following the Fig. 1. The samples marked with * show incongruent placement between the two phylogenetic reconstructions.

Both the mitochondrial *cox*1 and the chloroplast *rbc*L*-rbc*S sequences showed a noticeable level of polymorphism among the 102 samples of *D. fasciola* and *D. mediterranea* analysed in this study. Specifically, 60 and 46 variable sites were observed for the mtDNA (584 bp) and the cpDNA (510 bp) markers, respectively. The phylogenetic reconstructions obtained from these DNA regions (Fig. 2; Figs. S2–S3) inferred the existence of several highly supported monophyletic lineages (PP > 0.95) within the complex of *D. fasciola* and *D. mediterranea*. The analysed specimens were not clustered in two clades according to their taxonomic assignation, but subdivided in multiple nested lineages which did not correspond to a clear-cut differentiation between both species. While some of these lineages were exclusively constituted by specimens of one of the species, a few comprised samples of both *D. fasciola* and *D. mediterranea* intermixed. In particular, early diverging clades of the trees were mainly constituted by *D. mediterranea* specimens (with a few *D. fasciola* samples intermingled) while more derived clades were basically composed of *D. fasciola* specimens (with one or two *D. mediterranea* samples admixed). Comparing the trees obtained from mtDNA (Fig. 2A) and cpDNA (Fig. 2B), their topology showed overall congruence, except for a few (i.e., four out of 102) samples which appeared in non-equivalent clades. From a geographic point of view, the Atlantic specimens of *D. fasciola* were all clustered in highly derived clades of both the trees inferred from mtDNA and cpDNA markers. However, these derived clades also contained several samples from the Mediterranean Sea, including a few representatives of *D. mediterranea*. The trees inferred from both phylogenetic approaches (Bayesian and ML) resulted on congruent evolutionary reconstructions.

### Genetic variability of *D. fasciola* and *D. mediterranea*

**Figure 3:**
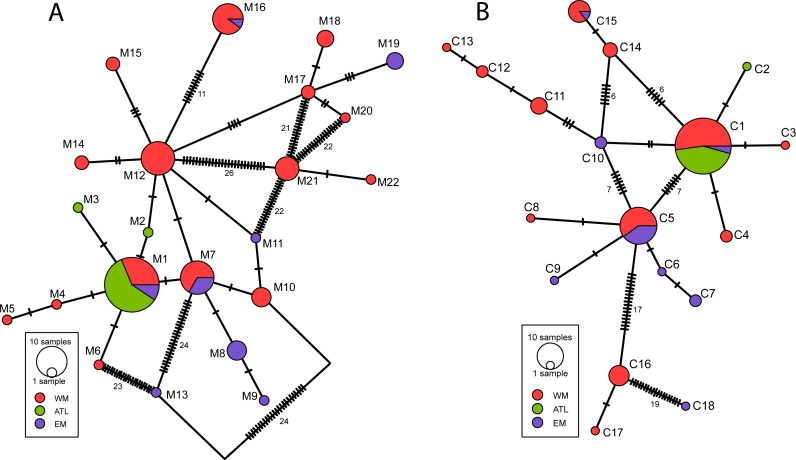
Minimum spanning network representing the haplotypes of *D. fasciola* and *D. mediterranea* sampling inferred from (A) *cox*1 and (B) *rbc*L-*rbc*S markers. Black stripes represent un-sampled intermediate haplotypes, one base mutation distant. The size of the circles represents the number of individuals and the color indicates their geographic circumscription. The number of unsampled mutation steps are shown when there are more than 5.

The number of haplotypes found in our study was 22 for *cox*1 region and 18 for *rbc*L-*rbc*S region. The minimum spanning networks of both markers revealed a similarly complex evolutionary structure (Fig. 3), with some groups of closely related haplotypes (connected by one-two mutation steps) loosely distanced to other groups of haplotypes (>3 mutation steps). The geographic distribution of the haplotypes among the different regions did not show a clear pattern. Only the two (cpDNA) or three (mtDNA) haplotypes present on the Atlantic region were all closely related among them, whereas those from the Western and Eastern Mediterranean appeared distributed all over the network. As occurred on the phylogenetic trees, the haplotype networks did not show a simple taxonomic pattern congruent with a clear differentiation involving two species (Fig. S1).

The result of genetic variability analyses is summarized in Table 2. Haplotype diversity (Hd) values was slightly higher for *cox*1 than for *rbc*L-*rbc*S, while nucleotide diversity (*π*) was very similar among the chloroplast and the mitochondrial regions. From a phylogeographic point of view, the samples from the Mediterranean Sea contained higher genetic variability—in terms of number of haplotypes, haplotype diversity and nucleotide diversity—than those from the Atlantic Ocean (Table 2). Haplotype richness calculated after rarefaction R_(18)_ was also several times higher in each of the Mediterranean groups than in the Atlantic one. Regarding the genetic variability within the Mediterranean groups, the Western samples showed more haplotypes (15 and 12 for mtDNA and cpDNA, respectively) than the Eastern ones (eight haplotypes for both mtDNA and cpDNA). However, the rest of genetic diversity indexes resulted in similar values among both regions of the Mediterranean Sea. In all cases, the results derived from both the mitochondrial and the chloroplast markers yielded congruent patterns of genetic variability.

**Table 2:** Genetic variability values for each molecular marker in the geographical groups of populations defined in the study.

	#P	N	*cox*1				*rbc*L-*rbc*S			
			Hp	Hd	R_(18)_	*π*	Hp	Hd	R_(18)_	*π*
Western Mediterranean	11	63	15	0.897	8.05	0.0175	12	0.804	6.27	0.0175
Eastern Mediterranean	9	18	8	0.882	7.00	0.0128	8	0.797	7.00	0.0131
Atlantic	12	21	3	0.186	1.71	0.0003	2	0.095	0.86	0.0002
Total	32	102	22	0.862	9.12	0.0142	18	0.753	7.14	0.0150

**Notes.**

#P number of sampling sites N number of individuals Hp number of haplotypes; Hd, haplotype diversity R(18) allelic richness after rarefaction *π* nucleotide diversity

## Discussion

### Systematic and taxonomic implications

Bayesian inference and ML trees show the occurrence of several statistically supported groups within *D. fasciola*–*D. mediterranea* complex, which do not seem to correspond to a clear-cut differentiation between the two species. Our data indicate that this group of *Dictyota* harbours more genetic diversity and complexity than previously envisaged. Earlier phylogenetic studies by Tronholm et al. (2010) and Tronholm et al. (2012) analysed several specimens of both species, which were placed in two independent clades in agreement with the taxonomic assignation of the samples. In our study, the expanded sampling along the distribution range of *D. fasciola* and *D. mediterranea*, together with the use of more variable markers, reveal additional lineages structured in a nested topology, which rejects a simple scenario with two monophyletic species.

Additionally, as explained above, several lineages in our phylogenetic reconstructions (Fig. 2) are constituted by samples of both species intermixed. These results may suggest that *D. fasciola* and *D. mediterranea* should not be segregated into the current two taxonomic units, but they could constitute a larger complex of cryptic species. Alternatively, the observed diversity could correspond to a single morphologically variable species, as already proposed by Feldmann (1937). There are well-documented examples of *Dictyota* species showing considerable morphological plasticity (e.g., *Dictyota ciliolata* Sonder ex Kützing, Tronholm et al. (2013); *Dictyota dichotoma* (Hudson) J.V.Lamouroux, Tronholm et al. (2008) so this could also be the case in the *D. fasciola-D. mediterranea* complex. The concordance among the trees derived from loci located in separate compartments of the genome (i.e., cpDNA and mtDNA; Fig. 2) suggests that this phylogenetic pattern—which disagrees with taxonomic delimitation—is not the product of incomplete lineage sorting processes (Leliaert et al., 2014).

The only possibility to accept the two currently recognized species would imply considering *D. mediterranea* as a paraphyletic taxon. Anacladogenetic speciation processes—often rendering ancestral paraphyletic taxa—have already been proposed to explain similar phylogenetic patterns (e.g., Hörandl, 2006; Crawford, 2010; Kuchta, Brown & Highton, 2018; Smith, Ooi & Clark, 2018). In this scenario, some taxonomy-genetic conflict should be taken into account: a few individuals of both species are nested with individuals of the other species (Fig. 2). Most of these cases occurs in sampling sites where the two species cohabit (e.g., Banyuls-sur-mer, in France; or Ladiko Bay, in Rhodes), so misplacements could potentially be caused by identification problems. However, a careful taxonomic determination was performed on each collected individual. Considering as well the close evolutionary relationship within the members of this group, we speculate that hybridization/introgression events between the different lineages are more likely explanations for these cases of taxonomy-genetic discordance. Future studies encompassing more comprehensive sampling, nuclear variable markers and thorough morphological analyses should be undertaken to disentangle the taxonomy of this *Dictyota* complex.

### Phylogeography and diversification within *D. fasciola*–*D. mediterranea* complex

The hypothesis formulated by Tronholm et al. (2010) to explain the diversification of *D. fasciola* and *D. mediterranea* complex do not fit well with the phylogeographic and genetic differentiation results obtained in our study. The scenario proposed by these authors considered that this group of seaweeds would have an Atlantic origin, colonizing the Mediterranean Sea posteriorly to the MSC. However, the genetic diversity values (Table 2) and the haplotype networks (Fig. 3) unambiguously show that the Mediterranean Sea contains much higher genetic diversity than the Atlantic Ocean. Similarly, the phylogenetic trees indicate that the early diverging lineages are always constituted by the Mediterranean specimens, whereas Atlantic samples are all clustered in a single younger lineage, indicating the derived character of the Atlantic distribution (Fig. 2). Even admitting that our sampling in the Atlantic Ocean is considerably incomplete, the extremely low genetic variability (Table 2) found among sampling sites distanced by several hundred kilometres results striking. These combined phylogeographic evidences suggest that the Mediterranean Sea could be the source area of diversification of the *D. fasciola*–*D. mediterranea* complex.

According to the time-calibrated phylogeny of the genus by Tronholm et al. (2012), this divergence process could have predated the MSC. By that time, the Mediterranean Sea showed a great geographical complexity, with some sub-basins mainly isolated among them (Piller, Harzhauser & Mandic, 2007). Surviving the MSC in these isolated Mediterranean refugia may have been accompanied by a reduction of population sizes, thereby enhancing divergence in allopatry of the isolated populations (Hörandl & Stuessy, 2010; Calvo et al., 2015). This scenario could explain the notably genetic differentiation observed within the *D. fasciola–D. mediterranea* complex in the Mediterranean Sea, as well as the low variability present in the Atlantic Ocean, which would have been putatively colonized after the reopening of the Gibraltar Strait connection. Although our phylogenetic reconstructions are not time-calibrated, our results clearly show that the colonization of the Atlantic Ocean occurred late in the diversification history of the complex.

However, as in the case of other Mediterranean organisms (e.g., vertebrates Domingues et al., 2005; echinoderms, Taboada & Pérez-Portela, 2016; or cnidarian, Pilczynska et al., 2017) we cannot discard the hypothesis that the ancestors of the *D. fasciola-D.mediterranea* complex survived the MSC in the Atlantic Ocean. In this scenario, the arrival of this group of seaweeds to the Mediterranean basin would have happened after the Zanclean re-flooding with Atlantic waters. Assuming the genetic drift occurring at the wave front of an expanding population (Excoffier & Ray, 2008), this phenomenon should have led to higher genetic diversity in Atlantic populations compared to the Mediterranean ones (i.e., exactly the opposite of what was observed in our results). To fit this hypothesis to the low genetic diversity and the derived phylogenetic position of the Atlantic samples found in our study, we should assume the subsequent extinction of most of the relict oceanic diversity after the colonization of the Mediterranean. Several studies have stated that Pleistocene glacial cycles erased Atlantic populations of marine organisms, while the isolated Mediterranean Sea offered a more stable persistence for some of them (e.g., Alberto et al., 2008; Lowe et al., 2012). The habitat fragmentation occurring in the Mediterranean during colder marine regression periods could have further enhanced genetic differentiation processes in this region (e.g., Arnaud-Haond et al., 2007; Rastorgueff et al., 2014). Therefore, a postglacial colonization of the Atlantic from Mediterranean sources would be an alternative or complementary explanation for phylogeographical patterns observed on *D. fasciola*–*D. mediterranea* complex.

## Conclusions

Our results indicate that *D. fasciola* and *D. mediterranea* are not monophyletic species. Conversely, we inferred a complex phylogenetic history challenging previous taxonomic and evolutionary hypotheses on this group of macroalgae. This study also highlights the key role played by the Mediterranean Sea as a refugium for these seaweeds during the major climatic changes occurred since the Miocene in this region of the planet. The limited number of sampling sites included in our study and the fact that some analysed populations consisted of few individuals prevent establishing more detailed phylogeographic hypotheses. Hence, more research focusing on this *Dictyota* complex—as well as on other algal groups—is needed to unravel the precise evolutionary and biogeographic response of seaweeds to the geological and climatic events that the Mediterranean experienced during and after the Messinian.

##  Supplemental Information

10.7717/peerj.6916/supp-1 Figure S1 Maximum likelihood tree inferred from *cox* 1 sequence data. Bootstrap support values, expressed as percentages, are given along branches

10.7717/peerj.6916/supp-2 Figure S2 Maximum likelihood tree inferred from* rbc* L-*rbc* S sequence data. Bootstrap support values, expressed as percentages, are given along branches

10.7717/peerj.6916/supp-3 Figure S3 Minimum spanning network representing the haplotypes of *D. fasciola* and *D. mediterranea* sampling inferred from (A) *cox1* and (B) *rbcL-rbcS* markers, with the colors indicating their taxonomic assignation Black stripes represent un-sampled intermediate haplotypes, one base mutation distant. The size of the circles represents the number of individuals. The number of unsampled mutation steps are shown when there are more than 5.

10.7717/peerj.6916/supp-4 Table S1 Geographic coordinates of *D. fasciola* and *D. mediterranea* specimens included in the study and Genbank accession numbers of each of the mtDNA and cpDNA regions sequenced

## Additional Information and Declarations

### Data Availability

The following information was supplied regarding data availability: The cox1 and rbcL-rbcS sequences are available at GenBank (accession numbers MH991431 to MH991636).

## References

[ref-1] Alberto F, Massa S, Manent P, Diaz-Almela E, Arnaud-Haond S, Duarte CM, Serrao EA (2008). Genetic differentiation and secondary contact zone in the seagrass *Cymodocea nodosa* across the Mediterranean–Atlantic transition region. Journal of Biogeography.

[ref-2] Aragay J, Vitales D, Gomez Garreta A, Ribera Siguan MA, Steen F, De Clerck O, Garnatje T, Rull Lluch J (2017). Phenological and molecular studies on the introduced seaweed *Dictyota cyanoloma* (Dictyotales, Phaeophyceae) along the Mediterranean coast of the Iberian Peninsula. Mediterranean Marine Science.

[ref-3] Arnaud-Haond S, Migliaccio M, Diaz-Almela E, Teixeira S, Van De Vliet MS, Alberto F, Procaccini G, Duarte CM, Serrao EA (2007). Vicariance patterns in the Mediterranean Sea: east–west cleavage and low dispersal in the endemic seagrass *Posidonia oceanica*. Journal of Biogeography.

[ref-4] Bandelt HJ, Forster P, Röhl A (1999). Median-joining networks for inferring intraspecific phylogenies. Molecular Biology and Evolution.

[ref-5] Boudouresque CF (2004). Marine biodiversity in the Mediterranean: status of species, populations and communities. Travaux scientifiques du Parc national de Port-Cros = Scientific reports of the Port-Cros National Park.

[ref-6] Bowen BW, Gaither MR, DiBattista JD, Iacchei M, Andrews KR, Grant WS, Toonen RJ, Briggs JC (2016). Comparative phylogeography of the ocean planet. Proceedings of the National Academy of Sciences of the United States of America.

[ref-7] Brodie J, Lewis J (2007). Unravelling the algae: the past, present, and future of algal systematics. The Systematics Association.

[ref-8] Calvo M, Alda F, Oliverio M, Templado J, Machordom A (2015). Surviving the Messinian Salinity Crisis? Divergence patterns in the genus *Dendropoma* (Gastropoda: Vermetidae) in the Mediterranean Sea. Molecular Phylogenetics and Evolution.

[ref-9] Coll M, Piroddi C, Steenbeek J, Kaschner K, Lasram FBR, Aguzzi J, Danovaro R (2010). The biodiversity of the Mediterranean Sea: estimates, patterns, and threats. PLOS ONE.

[ref-10] Coppejans E (1983). Iconographie d’algues Méditerranées. Chlorophyta, Phaeophyta, Rhodophyta. Bibliotheca Phycologica.

[ref-11] Cormaci M, Furnari G, Catra M, Alongi G, Giaccone G (2012). Flora marina bentonica del Mediterraneo: Phaeophyceae. Bollettino dell’Accademia Gioenia.

[ref-12] Crawford DJ (2010). Progenitor-derivative species pairs and plant speciation. Taxon.

[ref-13] Cullings KW (1992). Design and testing of a plant-specific PCR primer for ecological and evolutionary studies. Molecular Ecology.

[ref-14] Dayrat B (2005). Towards integrative taxonomy. Biological Journal of the Linnean Society.

[ref-15] Degnan JH, Rosenberg NA (2009). Gene tree discordance, phylogenetic inference and the multispecies coalescent. Trends in Ecology & Evolution.

[ref-16] Domingues VS, Bucciarelli G, Almada VC, Bernardi G (2005). Historical colonization and demography of the Mediterranean damselfish, *Chromis chromis*. Molecular Ecology.

[ref-17] Doyle JJ, Doyle JL (1987). A rapid DNA isolation procedure for small quantities of fresh leaf tissue. Phytochemical Bulletin.

[ref-18] Excoffier L, Ray N (2008). Surfing during population expansions promotes genetic revolutions and structuration. Trends in Ecology & Evolution.

[ref-19] Feldmann J (1937). Les algues marines de la côte des Albères. I–III. Cyanophycées, Chlorophycées, Phéophycées. Revue Algologique.

[ref-20] Guiry MD, Guiry GM (2019).

[ref-21] Hajibabaei M, Singer GA, Hebert PD, Hickey DA (2007). DNA barcoding: how it complements taxonomy, molecular phylogenetics and population genetics. Trends in Genetics.

[ref-22] Hörandl E (2006). Paraphyletic versus monophyletic taxa-evolutionary versus cladistic classifications. Taxon.

[ref-23] Hörandl E, Stuessy TF (2010). Paraphyletic groups as natural units of biological classification. Taxon.

[ref-24] Hsü KJ, Montadert L, Bernoulli D, Cita MB, Erickson A, Garrison RE, Kidd RB, Mèlierés F, Müller C, Wright R (1977). History of the Mediterranean salinity crisis. Nature.

[ref-25] Hu ZM, Duan DL, Lopez-Bautista J, Hu ZM, Fraser C (2016). Seaweed phylogeography from 1994 to 2014: an overview. Seaweed phylogeography.

[ref-26] Krijgsman W, Hilgen FJ, Raffi I, Sierro FJ, Wilson DS (1999). Chronology, causes and progression of the Messinian salinity crisis. Nature.

[ref-27] Kuchta SR, Brown AD, Highton R (2018). Disintegrating over space and time: paraphyly and species delimitation in the Wehrle’s Salamander complex. Zoologica Scripta.

[ref-28] Lanfear R, Frandsen PB, Wright AM, Senfeld T, Calcott B (2016). PartitionFinder 2: new methods for selecting partitioned models of evolution for molecular and morphological phylogenetic analyses. Molecular Biology and Evolution.

[ref-29] Leigh JW, Bryant D (2015). popart: full-feature software for haplotype network construction. Methods in Ecology and Evolution.

[ref-30] Leliaert F, Verbruggen H, Vanormelingen P, Steen F, López-Bautista JM, Zuccarello GC, De Clerck O (2014). DNA-based species delimitation in algae. European Journal of Phycology.

[ref-31] Lowe CD, Martin LE, Montagnes DJ, Watts PC (2012). A legacy of contrasting spatial genetic structure on either side of the Atlantic–Mediterranean transition zone in a marine protist. Proceedings of the National Academy of Sciences of the United States of America.

[ref-32] Maddison DR, Schulz KS, Maddison W (2007). The tree of life web project. Zootaxa.

[ref-33] Miller MA, Pfeiffer W, Schwartz T (2010). Creating the CIPRES Science Gateway for inference of large phylogenetic trees. Proceedings of the Gateway Computing Environments Workshop.

[ref-34] Motomura T, Nagasato C, Kimura K (2010). Cytoplasmic inheritance of organelles in brown algae. Journal of Plant Research.

[ref-35] Patarnello T, Volckaert FA, Castilho R (2007). Pillars of Hercules: is the Atlantic–Mediterranean transition a phylogeographical break?. Molecular Ecology.

[ref-36] Pena Martín C, Gómez Garreta A, Crespo MB (2004). Sobre la presencia de *Dictyota mediterranea* (Schiffner) G. Furnari (Dictyotales, Phaeophyceae) en la Península Ibérica. Acta Botanica Malacitana.

[ref-37] Petit RJ, El Mousadik A, Pons O (1998). Identifying populations for conservation on the basis of genetic markers. Conservation Biology.

[ref-38] Pezzolesi L, Falace A, Kaleb S, Hernandez-Kantun JJ, Cerrano C, Rindi F (2017). Genetic and morphological variation in an ecosystem engineer, *Lithophyllum byssoides* (Corallinales, Rhodophyta). Journal of Phycology.

[ref-39] Pilczynska J, Cocito S, Boavida J, Serrão EA, Queiroga H (2017). High genetic differentiation of red gorgonian populations from the Atlantic Ocean and the Mediterranean Sea. Marine Biology Research.

[ref-40] Piller WE, Harzhauser M, Mandic O (2007). Miocene Central Paratethys stratigraphy—current status and future directions. Stratigraphy.

[ref-41] Rastorgueff PA, Chevaldonné P, Arslan D, Verna C, Lejeusne C (2014). Cryptic habitats and cryptic diversity: unexpected patterns of connectivity and phylogeographical breaks in a Mediterranean endemic marine cave mysid. Molecular Ecology.

[ref-42] Reuschel S, Cuesta JA, Schubart CD (2010). Marine biogeographic boundaries and human introduction along the European coast revealed by phylogeography of the prawn *Palaemon elegans*. Molecular Phylogenetics and Evolution.

[ref-43] Ribera MA, Gómez-Garreta A, Gallardo T, Cormaci M, Furnari G, Giaccone G (1992). Check-list of Mediterranean Seaweeds. I. Fucophyceae (Warming 1884). Botanica Marina.

[ref-44] Ronquist F, Teslenko M, Van Der Mark P, Ayres DL, Darling A, Höhna S, Larget B, Liu L, Suchard M, Huelsenbeck J (2012). MrBayes 3.2: efficient Bayesian phylogenetic inference and model choice across a large model space. Systematic Biology.

[ref-45] Rozas J, Rozas R (1995). DnaSP, DNA sequence polymorphism: an interactive program for estimating population genetics parameters from DNA sequence data. Bioinformatics.

[ref-46] Schlick-Steiner BC, Steiner FM, Seifert B, Stauffer C, Christian E, Crozier RH (2010). Integrative taxonomy: a multisource approach to exploring biodiversity. Annual Review of Entomology.

[ref-47] Smith JF, Ooi MTY, Clark JL (2018). Incipient speciation in a neotropical Gesneriaceae: *Columnea kucyniakii* is nested within *C. strigosa*. Plant Systematics and Evolution.

[ref-48] Soltis DE, Soltis PS, Collier TG, Edgerton ML (1991). Chloroplast DNA variation within and among genera of the *Heuchera* group (Saxifragaceae): evidence for chloroplast transfer and paraphyly. American Journal of Botany.

[ref-49] Sotelo G, Morán P, Posada D (2009). Molecular phylogeny and biogeographic history of the European *Maja* spider crabs (Decapoda, Majidae). Molecular Phylogenetics and Evolution.

[ref-50] Stamatakis A (2006). Phylogenetic models of rate heterogeneity: a high performance computing perspective.

[ref-51] Stamatakis A (2014). RAxML version 8: a tool for phylogenetic analysis and post-analysis of large phylogenies. Bioinformatics.

[ref-52] Taboada S, Pérez-Portela R (2016). Contrasted phylogeographic patterns on mitochondrial DNA of shallow and deep brittle stars across the Atlantic-Mediterranean area. Scientific Reports.

[ref-53] Taviani M (2002). The Mediterranean benthos from late Miocene up to present: ten million years of dramatic climatic and geologic vicissitudes. Biologia Marina Mediterranea.

[ref-54] Thompson JD, Higgins DG, Gibson TJ (1994). CLUSTAL W: improving the sensitivity of progressive multiple sequence alignment through sequence weighting, position-specific gap penalties and weight matrix choice. Nucleic Acids Research.

[ref-55] Tronholm A, Afonso-Carrillo J, Sansón M, Leliaert F, Fernández-García C, De Clerck O (2013). Taxonomy of the *Dictyota ciliolata*–*crenulata* complex (Dictyotales, Phaeophyceae). Phycologia.

[ref-56] Tronholm A, Leliaert F, Sansón M, Afonso-Carrillo J, Tyberghein L, Verbruggen H, De Clerck O (2012). Contrasting geographical distributions as a result of thermal tolerance and long-distance dispersal in two allegedly widespread tropical brown algae. PLOS ONE.

[ref-57] Tronholm A, Sanson M, Afonso-Carrillo J, De Clerck O (2008). Distinctive morphological features, life-cycle phases and seasonal variations in subtropical populations of *Dictyota dichotoma* (Dictyotales, Phaeophyceae). Botanica Marina.

[ref-58] Tronholm A, Steen F, Tyberghein L, Leliaert F, Verbruggen H, Ribera Siguan MA, De Clerck O (2010). Species delimitation, taxonomy, and biogeography of *Dictyota* in Europe (Dictyotales, Phaeophyceae). Journal of Phycology.

[ref-59] Valsecchi E, Pasolini P, Bertozzi M, Garoia F, Ungaro N, Vacchi M, Sabelli B, Tinti F (2005). Rapid Miocene–Pliocene dispersal and evolution of Mediterranean rajid fauna as inferred by mitochondrial gene variation. Journal of Evolutionary Biology.

[ref-60] Verbruggen H (2014). Morphological complexity, plasticity, and species diagnosability in the application of old species names in DNA-based taxonomies. Journal of Phycology.

[ref-61] Will KW, Mishler BD, Wheeler QD (2005). The perils of DNA barcoding and the need for integrative taxonomy. Systematic Biology.

[ref-62] Xavier R, Zenboudji S, Lima FP, Harris DJ, Santos AM, Branco M (2011). Phylogeography of the marine isopod *Stenosoma nadejda* (Rezig, 1989) in North African Atlantic and western Mediterranean coasts reveals complex differentiation patterns and a new species. Biological Journal of the Linnean Society.

