# Supplementary figures and images for: Phylogeography of Dictyota fasciola and Dictyota mediterranea (Dictyotales, Phaeophyceae): unexpected patterns on the Atlantic-Mediterranean marine transition and taxonomic implications
Source: PeerJ. 2019 May 16;7:e6916. doi: 10.7717/peerj.6916 (PMC6526009; doi:10.7717/peerj.6916)

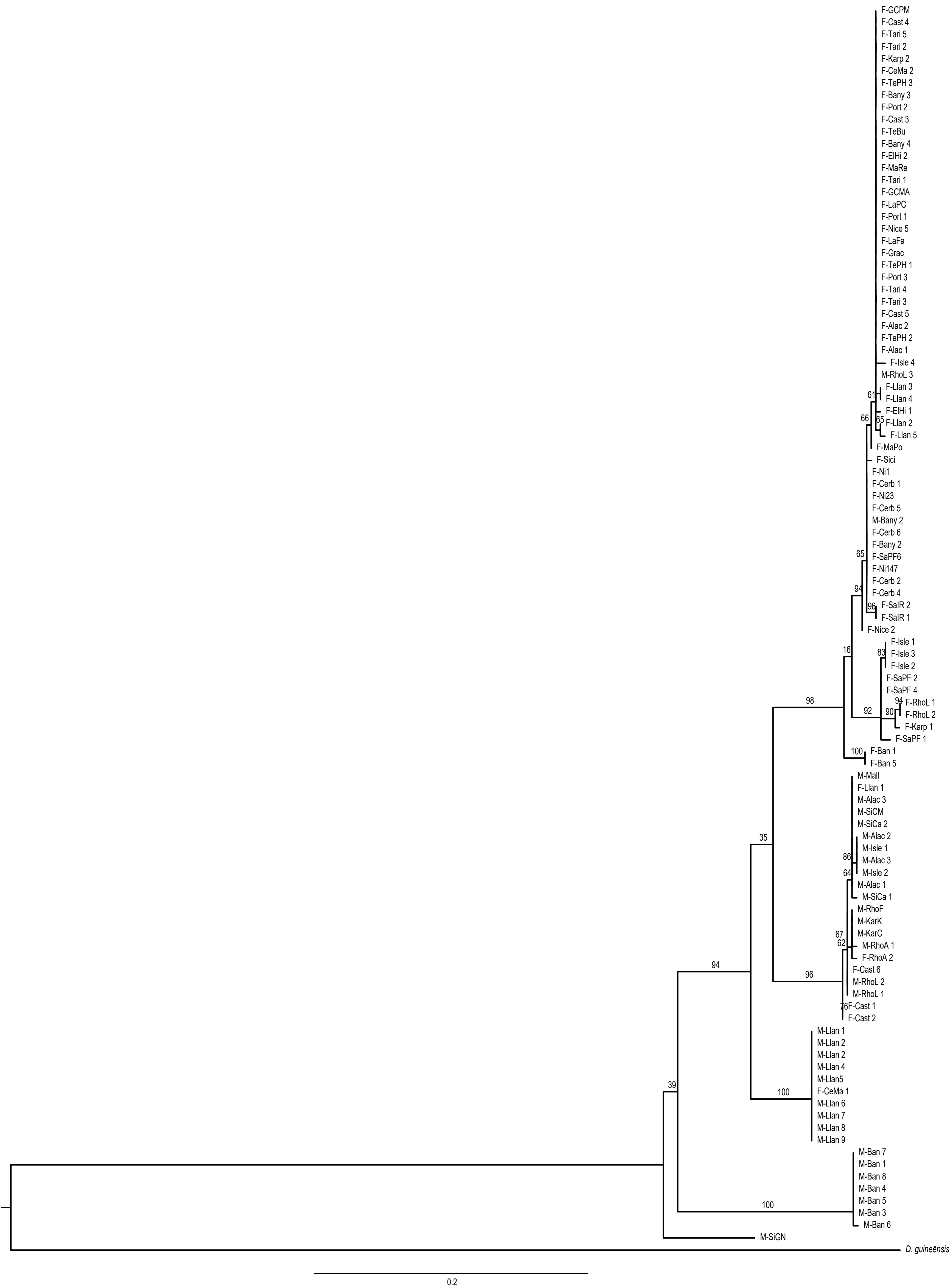

Supplement: Figure S1 [file peerj-07-6916-s001.pdf]

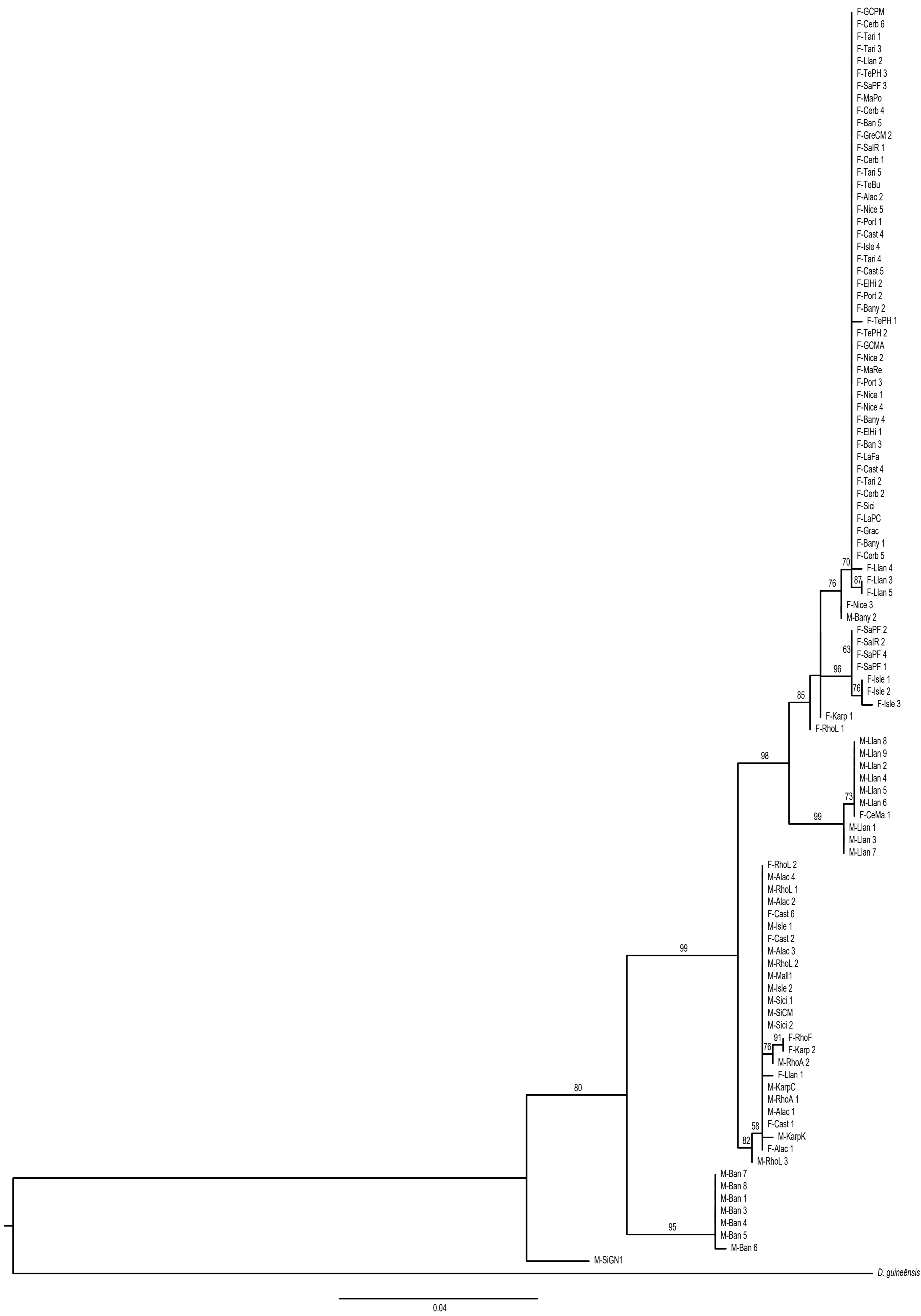

Supplement: Figure S2 [file peerj-07-6916-s002.pdf]
