# Supplementary material for: Phylogeography of Dictyota fasciola and Dictyota mediterranea (Dictyotales, Phaeophyceae): unexpected patterns on the Atlantic-Mediterranean marine transition and taxonomic implications
Source: PeerJ. 2019 May 16;7:e6916. doi: 10.7717/peerj.6916 (PMC6526009; doi:10.7717/peerj.6916)
Supplement: Table S1 [file peerj-07-6916-s004.docx]

Table S1. Specimens of *D. fasciola* and *D. mediterranea* included in the study and Genbank accession numbers of each of the mtDNA and cpDNA regions sequenced.

|  |  | Genbank  accession numbers | | Geographic coordinates |
| --- | --- | --- | --- | --- |
| Species | Code ind. | *Cox*1 | *rbc*L-*rbc*S |  |
| *Dictyota fasciola* | F-Alac_1 | MH991569 | MH991431 | 38°21'18.8"N 0°25'37.7"W |
|  | F-Alac_2 | MH991570 | MH991432 |  |
|  | F-Bany_1 | MH991571 | MH991433 | 42°28'55.0"N 3°08'13.4"E |
|  | F-Bany_2 | MH991572 | MH991434 |  |
|  | F-Bany_3 | MH991573 | MH991435 |  |
|  | F-Bany_4 | MH991574 | MH991436 |  |
|  | F-Bany_5 | MH991575 | MH991437 |  |
|  | F-ElHi_1 | MH991576 | MH991438 | 27°38'18.0"N 17°59'05.6"W |
|  | F-ElHi_2 | MH991577 | MH991439 |  |
|  | F-LaFa | MH991578 | MH991440 | 29°07'09.3"N 13°33'48.6"W |
|  | F-LaPC | MH991579 | MH991441 | 28°55'08.2"N 13°39'58.7"W |
|  | F-Grac | MH991580 | MH991442 | 29°13'31.2"N 13°30'17.0"W |
|  | F-TePH_1 | MH991581 | MH991443 | 28°34'47.3"N 16°19'30.4"W |
|  | F-TePH_2 | MH991582 | MH991444 |  |
|  | F-TePH_3 | MH991585 | MH991447 |  |
|  | F-GCMA | MH991583 | MH991445 | 27°48'01.2"N 15°44'21.3"W |
|  | F-GCPM | MH991584 | MH991446 | 27°44'04.1"N 15°35'58.1"W |
|  | F-TeBu | MH991586 | MH991448 | 28°22'16.9"N 16°52'17.3"W |
|  | F-Cast_1 | MH991587 | MH991449 | 40°20'11.1"N 0°22'49.6"E |
|  | F-Cast_2 | MH991588 | MH991450 |  |
|  | F-Cast_3 | MH991589 | MH991451 |  |
|  | F-Cast_4 | MH991590 | MH991452 |  |
|  | F-Cast_5 | MH991591 | MH991453 |  |
|  | F-Cast_6 | MH991592 | MH991454 |  |
|  | F-Cerb_1 | MH991593 | MH991455 | 42°27'14.8"N 3°09'57.9"E |
|  | F-Cerb_2 | MH991594 | MH991456 |  |
|  | F-Cerb_3 | MH991595 | MH991457 |  |
|  | F-Cerb_4 | MH991596 | MH991458 |  |
|  | F-Cerb_5 | MH991597 | MH991459 |  |
|  | F-CeMa_1 | MH991598 | MH991460 | 40°05'53.0"N 23°58'54.1"E |
|  | F-CeMa_2 | MH991599 | MH991461 |  |
|  | F-Isle_1 | MH991600 | MH991462 | 36°48'50.6"N 2°03'03.1"W |
|  | F-Isle_2 | MH991601 | MH991463 |  |
|  | F-Isle_3 | MH991602 | MH991464 |  |
|  | F-Isle_4 | MH991603 | MH991465 |  |
|  | F-Karp_1 | MH991604 | MH991466 | 35°37'43.6"N 27°09'26.0"E |
|  | F-Karp_2 | MH991605 | MH991467 |  |
|  | F-Llan_1 | MH991606 | MH991468 | 42°23'7.80"N 3° 9'37.27"E |
|  | F-Llan_2 | MH991607 | MH991469 |  |
|  | F-Llan_3 | MH991608 | MH991470 |  |
|  | F-Llan_4 | MH991609 | MH991471 |  |
|  | F-Llan_5 | MH991610 | MH991472 |  |
|  | F-MaPo | MH991611 | MH991473 | 32°45'03.4"N 16°44'04.4"W |
|  | F-MaRe | MH991612 | MH991474 | 32°38'29.9"N 16°49'54.6"W |
|  | F-Nice_1 | MH991613 | MH991475 | 43°41'10.2"N 7°17'53.0"E |
|  | F-Nice_2 | MH991614 | MH991476 |  |
|  | F-Nice_3 | MH991615 | MH991477 |  |
|  | F-Nice_4 | MH991616 | MH991478 |  |
|  | F-Nice_5 | MH991617 | MH991479 |  |
|  | F-Port_1 | MH991618 | MH991480 | 37°49'37.9"N 8°47'31.9"W |
|  | F-Port_2 | MH991619 | MH991481 |  |
|  | F-Port_3 | MH991620 | MH991482 |  |
|  | F-RhoL_1 | MH991621 | MH991483 | 36°19'13.8"N 28°12'19.2"E |
|  | F-RhoL_2 | MH991622 | MH991484 |  |
|  | F-RhoF_ | MH991623 | MH991485 | 36°06'23.3"N 27°44'13.3"E |
|  | F-SaIR_1 | MH991624 | MH991486 | 41°00'52.6"N 8°52'13.7"E |
|  | F-SaIR_2 | MH991625 | MH991487 |  |
|  | F-SaPF_1 | MH991626 | MH991488 | 40°40'41.9"N 8°12'01.9"E |
|  | F-SaPF_2 | MH991627 | MH991489 |  |
|  | F-SaPF_3 | MH991628 | MH991490 |  |
|  | F-SaPF_4 | MH991629 | MH991491 |  |
|  | F-Sici | MH991630 | MH991492 | 37°33'14.5"N 15°09'01.3"E |
|  | F-Tari_1 | MH991631 | MH991493 | 36°00'28.4"N 5°36'26.5"W |
|  | F-Tari_2 | MH991632 | MH991494 |  |
|  | F-Tari_3 | MH991633 | MH991495 |  |
|  | F-Tari_4 | MH991634 | MH991496 |  |
|  | F-Tari_5 | MH991635 | MH991497 |  |
| *Dictyota mediterranea* | M-Alac_1 | MH991534 | MH991498 | 38°21'18.8"N 0°25'37.7"W |
|  | M-Alac_2 | MH991535 | MH991499 |  |
|  | M-Alac_3 | MH991536 | MH991500 |  |
|  | M-Alac_4 | MH991537 | MH991501 |  |
|  | M-Bany_1 | MH991538 | MH991502 | 42°28'55.0"N 3°08'13.4"E |
|  | M-Bany_2 | MH991539 | MH991503 |  |
|  | M-Bany_3 | MH991540 | MH991504 |  |
|  | M-Bany_4 | MH991541 | MH991505 |  |
|  | M-Bany_5 | MH991542 | MH991506 |  |
|  | M-Bany_6 | MH991543 | MH991507 |  |
|  | M-Bany_7 | MH991544 | MH991508 |  |
|  | M-Bany_8 | MH991545 | MH991509 |  |
|  | M-Isle_1 | MH991546 | MH991510 | 36°48'50.6"N 2°03'03.1"W |
|  | M-Isle_2 | MH991547 | MH991511 |  |
|  | M-KarK | MH991548 | MH991512 | 35°28'11.6"N 27°11'36.6"E |
|  | M-KarC | MH991549 | MH991513 | 35°26'56.7"N 27°09'41.7"E |
|  | M-Llan_1 | MH991550 | MH991514 | 42°23'7.80"N 3° 9'37.27"E |
|  | M-Llan_2 | MH991551 | MH991515 |  |
|  | M-Llan_3 | MH991552 | MH991516 |  |
|  | M-Llan_4 | MH991553 | MH991517 |  |
|  | M-Llan_5 | MH991554 | MH991518 |  |
|  | M-Llan_6 | MH991555 | MH991519 |  |
|  | M-Llan_7 | MH991556 | MH991520 |  |
|  | M-Llan_8 | MH991557 | MH991521 |  |
|  | M-Llan_9 | MH991558 | MH991522 |  |
|  | M-Mall | MH991559 | MH991523 | 39°45'49.3"N 3°09'43.5"E |
|  | M-RhoL_1 | MH991560 | MH991524 | 36°19'13.8"N 28°12'19.2"E |
|  | M-RhoL_2 | MH991561 | MH991525 |  |
|  | M-RhoL_3 | MH991562 | MH991526 |  |
|  | M-RhoA_1 | MH991563 | MH991527 | 36°04'29.4"N 28°02'54.2"E |
|  | M-RhoA_2 | MH991564 | MH991528 |  |
|  | M-Sici_1 | MH991565 | MH991529 | 37°33'14.5"N 15°09'01.3"E |
|  | M-Sici_2 | MH991566 | MH991530 |  |
|  | M-SiGN | MH991567 | MH991531 | 37°49'18.5"N 15°16'41.5"E |
|  | M-SiCM | MH991568 | MH991532 | 38°16'17.0"N 15°13'50.2"E |
| *Dictyota guinëensis* | G_3141 | MH991636 | MH991533 |  |
