# Supplementary material for: Phylogeography of Dictyota fasciola and Dictyota mediterranea (Dictyotales, Phaeophyceae): unexpected patterns on the Atlantic-Mediterranean marine transition and taxonomic implications
Source: PeerJ. 2019 May 16;7:e6916. doi: 10.7717/peerj.6916 (PMC6526009; doi:10.7717/peerj.6916)
Supplement: Figure S3 — Black stripes represent un-sampled intermediate haplotypes, one base mutation distant. The size of the circles represents the number of individuals. The number of unsampled mutation steps are shown when there are more than 5. [file peerj-07-6916-s003.pdf]

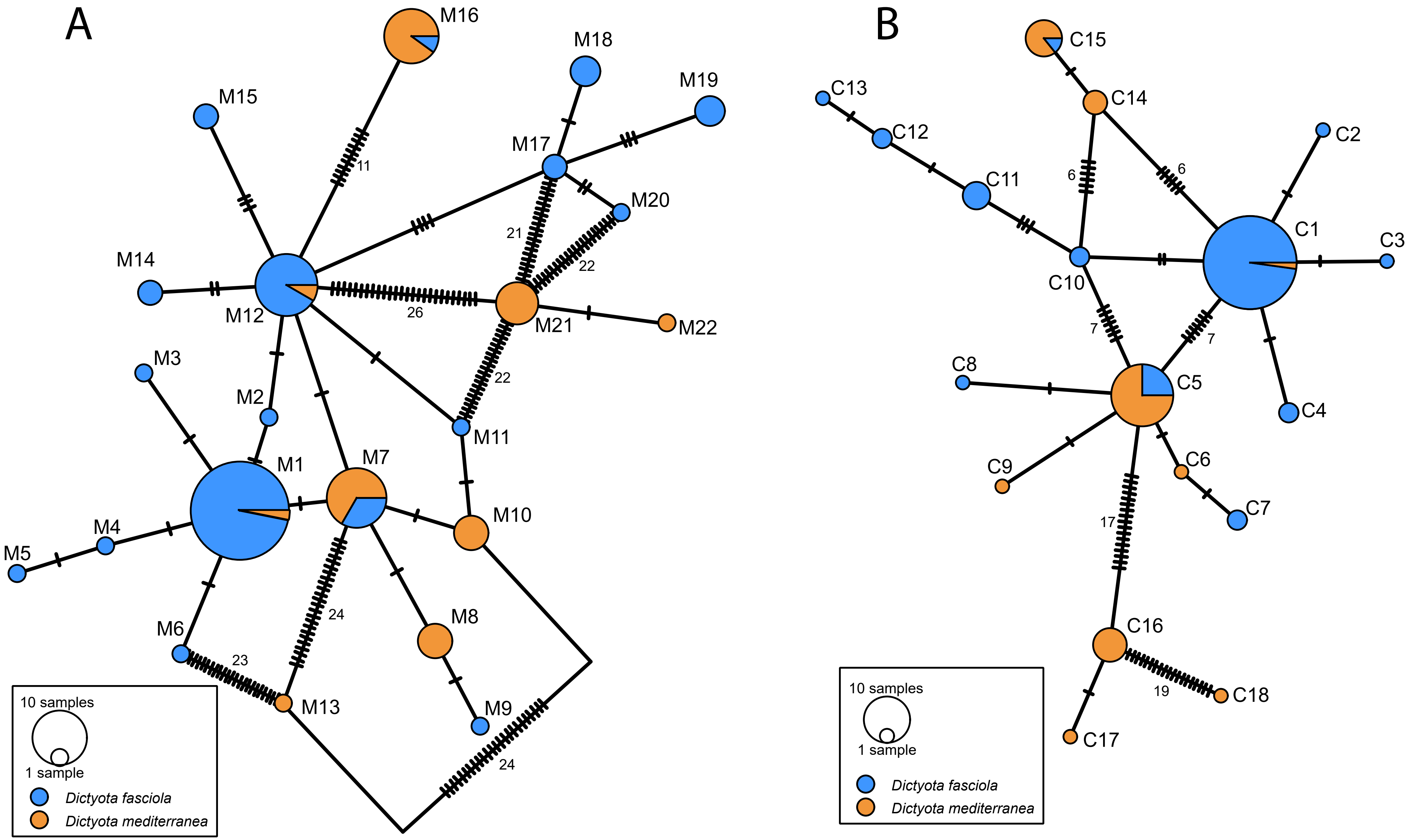

A
M1
M2
M3
M4
M5
M6
M7
M8
M9
M10
M11
M12
M13
M14
M15
M16
M17
M18
M19
M20
M21
M22
11
21
22
26
22
24
23
24
10 samples
1 sample
Dictyota fasciola
Dictyota mediterranea
B
C1
C2
C3
C4
C5
C6
C7
C8
C9
C10
C11
C12
C13
C14
C15
C16
C17
C18
6
6
7
7
17
19
10 samples
1 sample
Dictyota fasciola
Dictyota mediterranea

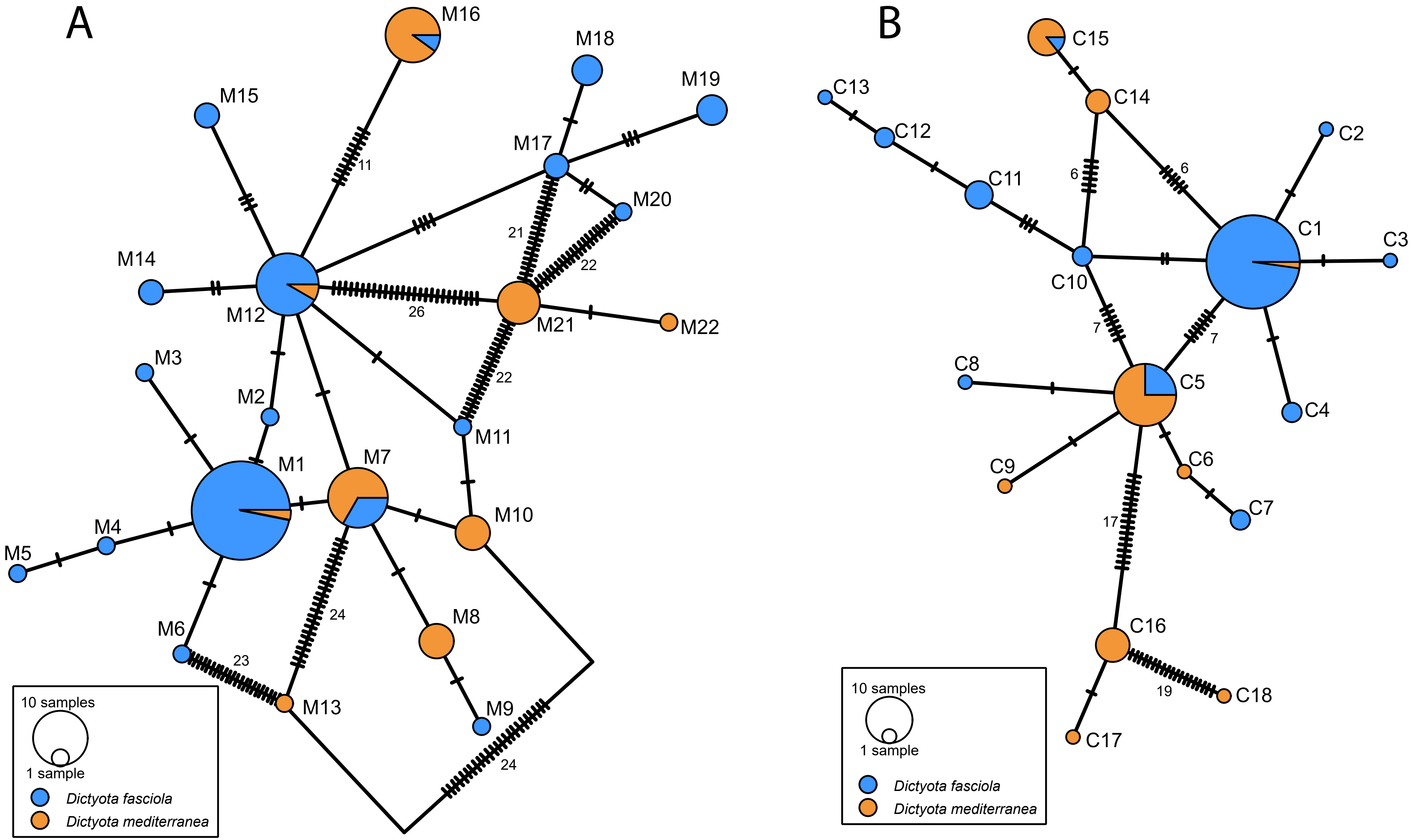
A
M1
M2
M3
M4
M5
M6
M7
M8
M9
M10
M11
M12
M13
M14
M15
M16
M17
M18
M19
M20
M21
M22
11
21
22
26
22
24
23
24
10 samples
1 sample
Dictyota fasciola
Dictyota mediterranea
B
C1
C2
C3
C4
C5
C6
C7
C8
C9
C10
C11
C12
C13
C14
C15
C16
C17
C18
6
6
7
7
17
19
10 samples
1 sample
Dictyota fasciola
Dictyota mediterranea
